# Tanshinone IIA inhibits angiogenesis in human endothelial progenitor cells *in vitro* and *in vivo*

**DOI:** 10.18632/oncotarget.22649

**Published:** 2017-11-24

**Authors:** Hsiang-Ping Lee, Yueh-Ching Liu, Po-Chun Chen, Huai-Ching Tai, Te-Mao Li, Yi-Chin Fong, Chih-Shiang Chang, Min-Huan Wu, Li-Pin Chiu, Chia-Jung Wang, Yi-Hsuan Chen, Yih-Jer Wu, Chih-Hsin Tang, Shih-Wei Wang

**Affiliations:** ^1^ Graduate Institute of Chinese Medicine, China Medical University, Taichung, Taiwan; ^2^ Department of Chinese Medicine, China Medical University Hospital, Taichung, Taiwan; ^3^ Department of Orthopaedics, Mackay Memorial Hospital, Taipei, Taiwan; ^4^ Graduate Institute of Biomedical Science, China Medical University, Taichung, Taiwan; ^5^ School of Medicine, Fu-Jen Catholic University, New Taipei City, Taiwan; ^6^ Department of Urology, Fu-Jen Catholic University Hospital, New Taipei City, Taiwan; ^7^ Department of Urology, National Taiwan University Hospital, Taipei, Taiwan; ^8^ Department of Sports Medicine, College of Health Care, China Medical University, Taichung, Taiwan; ^9^ Department of Orthopedic Surgery, China Medical University Beigang Hospital, Yun-Lin County, Taiwan; ^10^ Graduate Institute of Pharmaceutical Chemistry, China Medical University, Taichung, Taiwan; ^11^ Physical Education Office, Tunghai University, Taichung, Taiwan; ^12^ Sports Recreation and Health Management Continuing Studies, Tunghai University, Taichung, Taiwan; ^13^ Department of Nursing, Taipei City Hospital, Taipei, Taiwan; ^14^ General Education Center, University of Taipei, Taipei, Taiwan; ^15^ Department of Medicine, Mackay Medical College, New Taipei City, Taiwan; ^16^ Department of Pharmacology, School of Medicine, China Medical University, Taichung, Taiwan; ^17^ Department of Biotechnology, College of Health Science, Asia University, Taichung, Taiwan; ^18^ Graduate Institute of Natural Products, College of Pharmacy, Kaohsiung Medical University, Kaohsiung, Taiwan

**Keywords:** endothelial progenitor cells, angiogenesis, tanshinone IIA, VEGF-A

## Abstract

Accumulating evidence reports that bone marrow-derived endothelial progenitor cells (EPCs) regulate angiogenesis, postnatal neovascularization and tumor metastasis. It has been suggested that understanding the molecular targets and pharmacological functions of natural products is important for novel drug discovery. Tanshinone IIA is a major diterpene quinone compound isolated from Danshen (*Salvia miltiorrhiza*) and is widely used in traditional Chinese medicine (TCM). Evidence indicates that tanshinone IIA modulates angiogenic functions in human umbilical vein endothelial cells. However, the anti-angiogenic activity of tanshinone IIA in human EPCs has not been addressed. Here, we report that tanshinone IIA dramatically suppresses vascular endothelial growth factor (VEGF)-promoted migration and tube formation of human EPCs, without cytotoxic effects. We also show that tanshinone IIA markedly inhibits VEGF-induced angiogenesis in the chick embryo chorioallantoic membrane (CAM) model. Importantly, tanshinone IIA significantly attenuated microvessel formation and the expression of EPC-specific markers in the *in vivo* Matrigel plug assay in mice. Further, we found that tanshinone IIA inhibits EPC angiogenesis through the PLC, Akt and JNK signaling pathways. Our report is the first to reveal that tanshinone IIA reduces EPC angiogenesis both *in vitro* and *in vivo*. Tanshinone IIA is a promising natural product worthy of further development for the treatment of cancer and other angiogenesis-related pathologies.

## INTRODUCTION

Angiogenesis is a critical step in the physiology of tissue repair, bone remodeling and reproduction, as well as embryonic development [1]. Angiogenesis also plays an important role during pathological processes, including various inflammatory diseases and tumor progression, as well as metastasis [2-4]. Inhibiting angiogenesis is therefore a critical strategy in cancer therapy and other angiogenesis-related disorders [5, 6]. Currently, more than 10 angiogenesis inhibitors are in clinical use. Most target the vascular endothelial growth factor (VEGF) receptor (VEGFR) axis, using small molecule inhibitors or antibody agents [7, 8]. Since VEGF and its receptors are critical regulators of angiogenesis in tumor cells, VEGF and VEGFR signaling are promising therapeutic targets in cancer metastasis and treatment.

Bone marrow-derived endothelial progenitor cells (EPCs) contribute to postnatal physiological and pathological neovascularization [9]. EPCs have cellular subpopulations and contain different functional capacities such as migration, proliferation, and recruitment in response to angiogenesis. Circulating EPCs, a cell population characterized by the CD133/CD34/VEGFR2 phenotype, are mobilized from the bone marrow into the bloodstream and contribute to neovascularization by direct incorporation into neovessels [10]. Tumor-secreted growth factors, such as VEGF, control the mobilization of EPCs, which subsequently contribute to cancer growth and angiogenesis of certain tumors [11]. It has been suggested that EPCs act as critical regulators of the angiogenic switch that controls the development of micrometastasis and subsequently promotes tumor macrometastasis [12]. All of this evidence supports the role of EPCs in tumor angiogenesis and metastasis. Selective targeting of EPCs may have potential as an anti-angiogenic strategy against cancer metastasis.

Several natural products have been documented to reduce angiogenesis via different mechanisms [13-15]. Danshen, the dried root of *Salvia miltiorrhiza* Bunge, is a well-known herb in traditional Chinese medicine (TCM) that is mainly used clinically in preventative or therapeutic preparations for hepatitis, arthritis, stroke, vascular disease, coronary heart disease and cancer [16, 17]. Tanshinone IIA is a major diterpene extracted from danshen that possesses anti-atherosclerotic, anti-inflammatory and anti-oxidative effects [18-20]. Tanshinone IIA inhibits angiogenesis in human umbilical vein endothelial cells (HUVECs) [21, 22], but its anti-angiogenic effects in EPCs are largely unknown. In this study, we report that tanshinone IIA inhibits EPC migration and tube formation without any evidence of cytotoxic activity. Moreover, tanshinone IIA impedes angiogenesis and the expression of progenitor cell markers *in vivo*. Tanshinone IIA shows potential as a promising angiogenesis inhibitor that targets EPCs.

## RESULTS

### Tanshinone IIA shows no cytotoxicity in human EPCs

**Figure 1:**
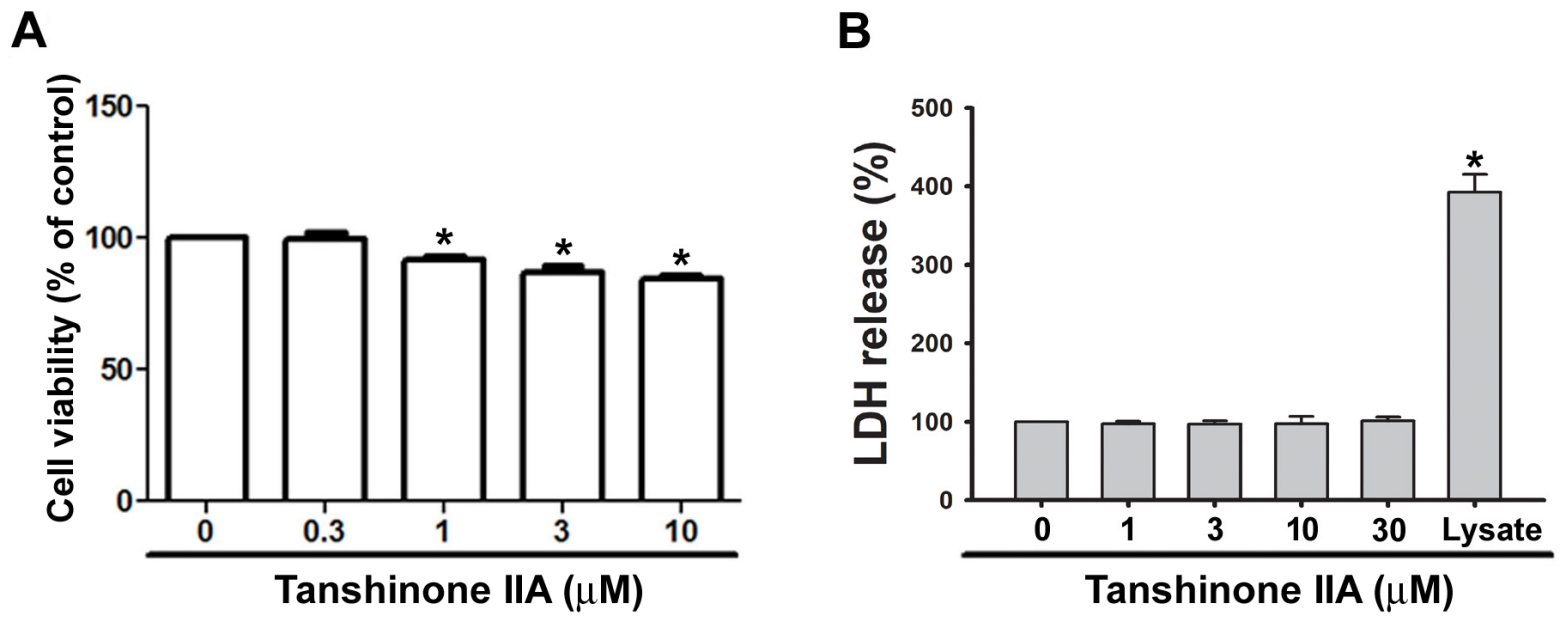
Effects of tanshinone IIA on cell growth and cytotoxicity in human EPCs EPCs were incubated with the indicated concentrations of tanshinone IIA for 24 h, then cell viability **(A)** and cytotoxicity **(B)** were determined using the MTT and LDH assays, respectively. Data represent the mean ± S.E.M. of four independent experiments. ^*^, *p* < 0.05 compared with the control group.

We first used the MTT assay to examine the effect of tanshinone IIA upon cell viability in EPCs. Tanshinone IIA (1-10 μM) slightly reduced cell growth of EPCs after 24 h of treatment (Figure 1A). To investigate whether this effect was caused by cytotoxicity, we performed the lactate dehydrogenase (LDH) assay in EPCs. As shown in Figure 1B, treatment with tanshinone IIA for 24 h did not induce LDH release, even at a high concentration of tanshinone IIA (30 μM). In addition, prolonged treatment of tanshinone IIA for 48 h did not induce LDH release in EPCs (Supplementary Figure 1). Therefore, we suggest that tanshinone IIA exhibits safe activity without causing cytotoxicity in human EPCs.

### Tanshinone IIA inhibits VEGF-induced migration and tube formation of EPCs

**Figure 2:**
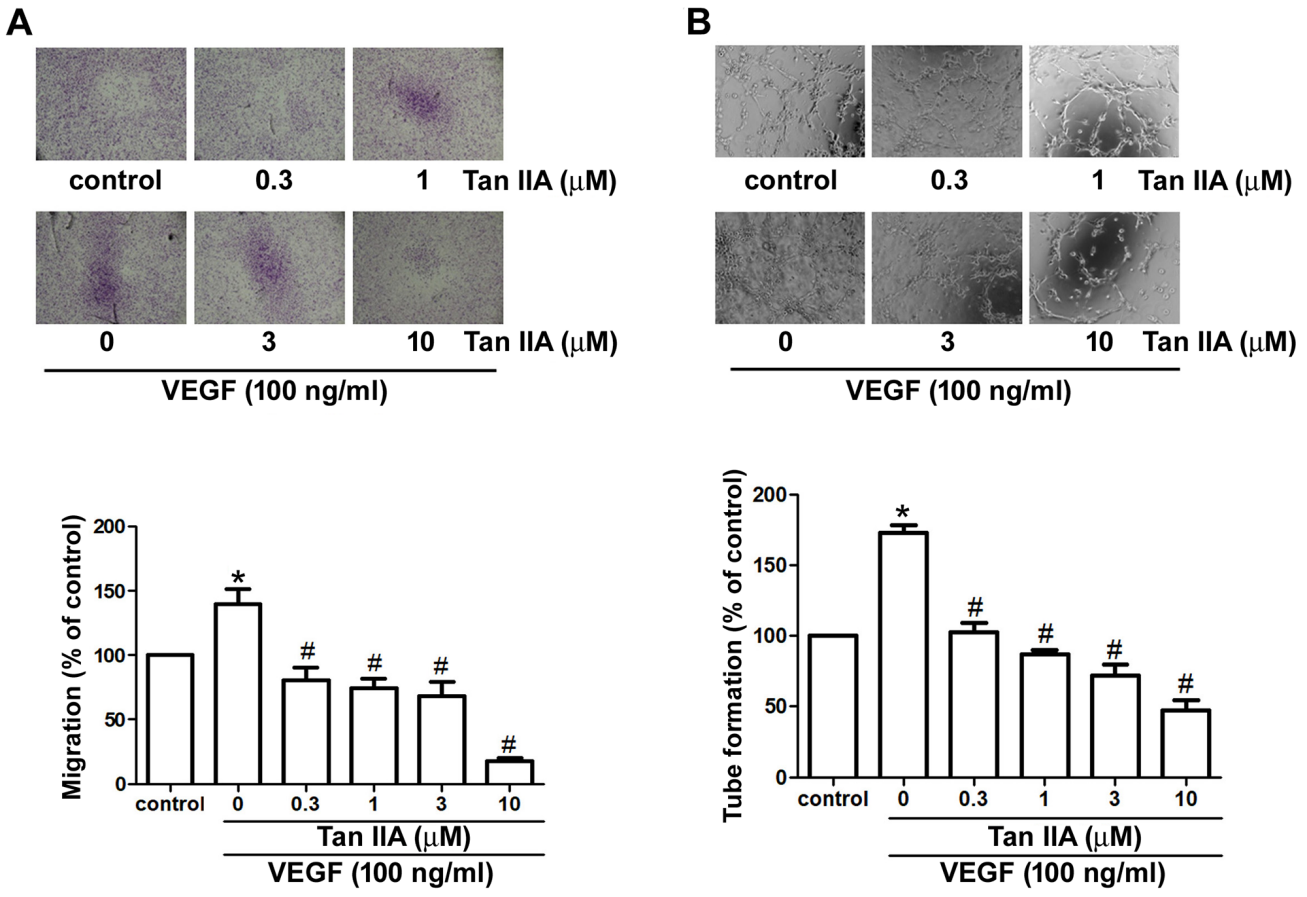
Effects of tanshinone IIA on VEGF-induced migration and tube formation in human EPCs EPCs were stimulated with or without VEGF (100 ng/ml) in the absence or presence of various concentrations of tanshinone IIA for 24 h. Cell migration **(A)** and capillary-like structure formation **(B)** were examined by Transwell and tube formation assays, respectively. Data represent the mean ± S.E.M. of four independent experiments. ^*^, *p* < 0.05 compared with the control group; ^#^, *p* < 0.05 compared with the VEGF-treated group.

VEGF is the most crucial angiogenic factor during physiological and pathological angiogenesis. Migration of EPCs through normal tissue boundaries (the basement membrane) is an important event in neovessel synthesis [30]. We used Transwell chambers to investigate the effects of tanshinone IIA on EPC migration. We found that tanshinone IIA inhibited VEGF-promoted migration in a concentration-dependent manner (Figure 2A). We next performed the tube formation assay to validate the anti-angiogenic activity of tanshinone IIA in EPCs. As shown in Figure 2B, VEGF stimulation resulted in EPC reorganization and formation of capillary-like structures. Tanshinone IIA significantly suppressed VEGF-induced tube formation of EPCs. Collectively, these results indicate that noncytotoxic concentrations of tanshinone IIA exhibit promising anti-angiogenic effects in human EPCs.

### Tanshinone IIA inhibits VEGF-induced PLC and Akt activation in EPCs

**Figure 3:**
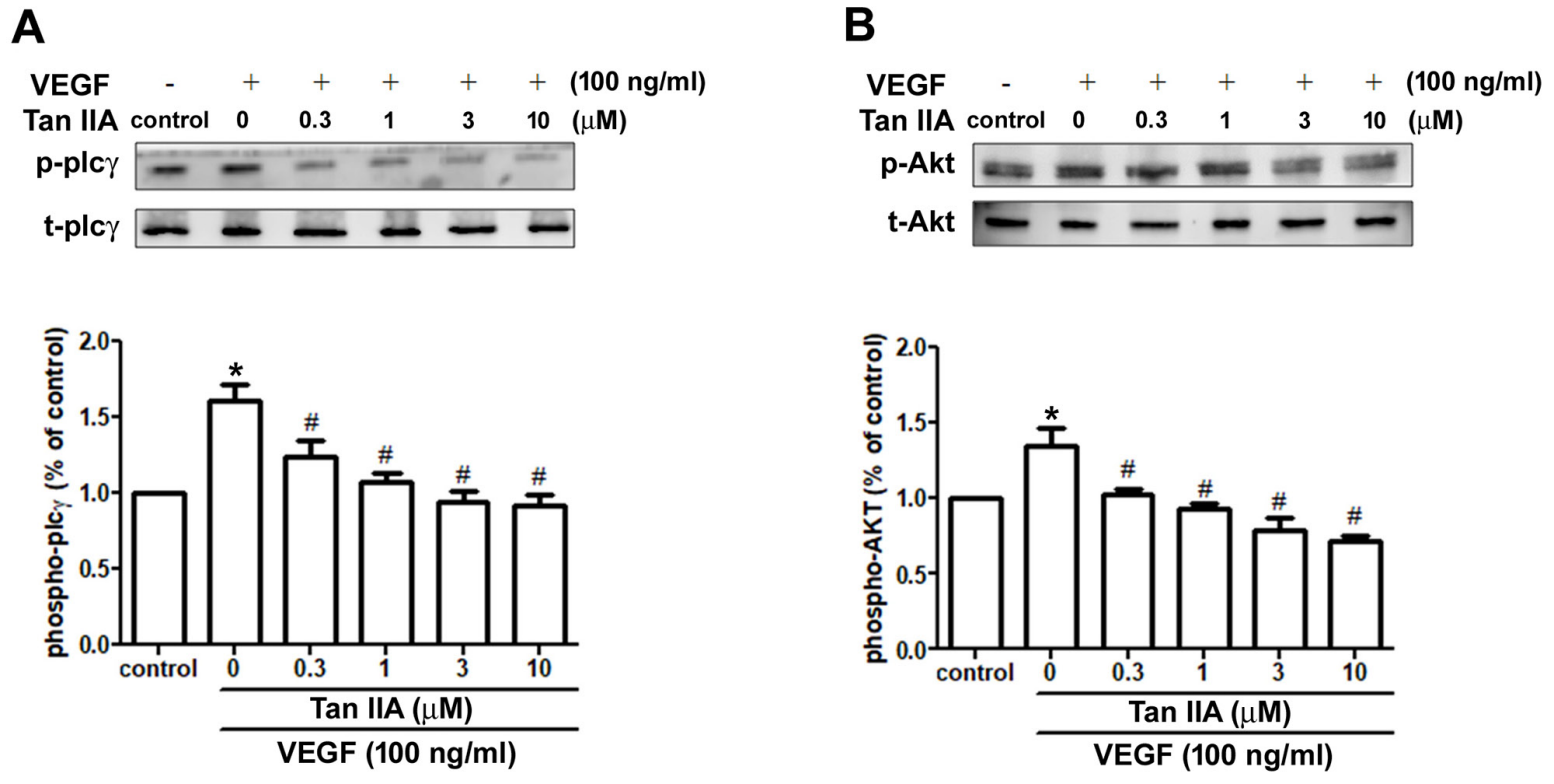
Effects of tanshinone IIA on PLC and Akt signaling pathways in human EPCs EPCs were incubated with VEGF-A (100 ng/ml) and the indicated concentrations of tanshinone IIA for 24 h. Phosphorylation of PLC **(A)** and Akt **(B)** was examined by Western blotting. Data represent the mean ± S.E.M. of four independent experiments. ^*^, *p* < 0.05 compared with the control group; ^#^, *p* < 0.05 compared with the VEGF-treated group.

PLC and Akt activation are required to facilitate angiogenesis in EPCs [31, 32]. We therefore examined whether the inhibitory effect of tanshinone IIA is due to its ability to interfere with VEGF-induced activation of the PLC and Akt pathways. Incubation of EPCs with VEGF promoted the phosphorylation of PLC and Akt, whereas VEGF-induced PLC and Akt phosphorylation was inhibited by tanshinone IIA treatment in a concentration-dependent manner (Figure 3). Thus, we suggest that tanshinone IIA may suppress VEGF-induced EPC angiogenesis via the PLC- and Akt-dependent pathways. Evidence indicates the involvement of the PDK1, PI3K and mTOR signaling pathways in Akt activation [33]. However, we found that tanshinone IIA did not affect PDK1, PI3K, or mTOR phosphorylation (Supplementary Figure 2). Whether there is some other signaling interaction with Akt needs to be investigated.

### Tanshinone IIA inhibits VEGF-induced JNK but not ERK and p38 phosphorylation in EPCs

**Figure 4:**
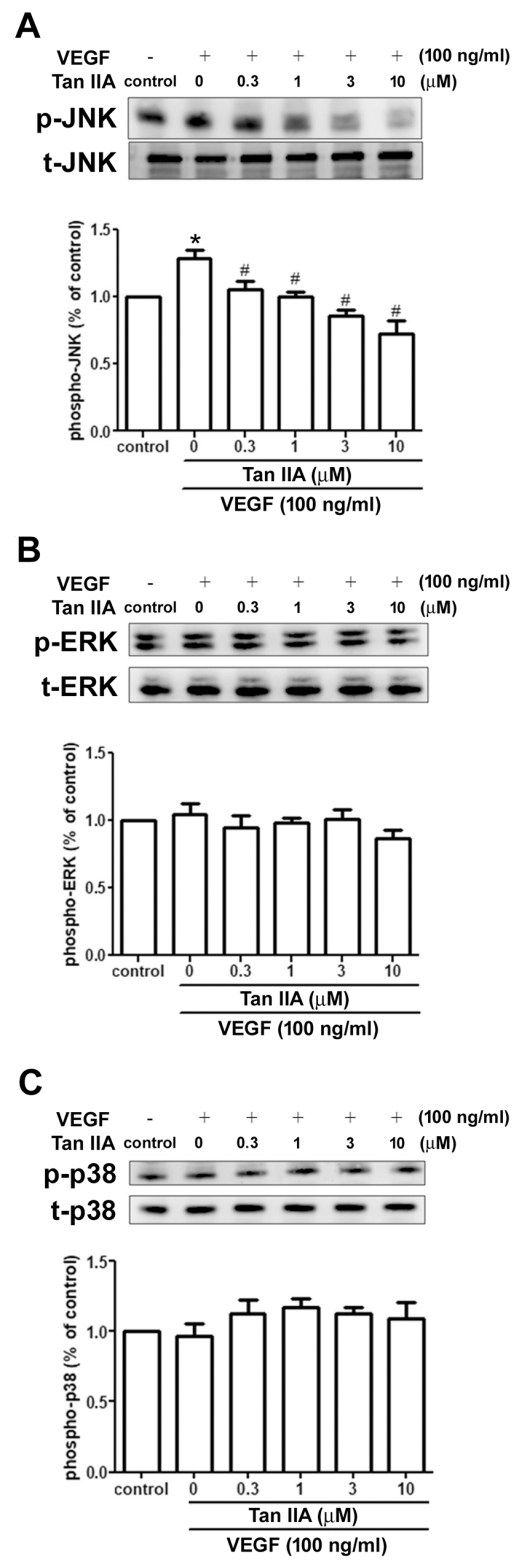
Effects of tanshinone IIA on the MAPK pathway in human EPCs EPCs were incubated with VEGF-A (100 ng/ml) and the indicated concentrations of tanshinone IIA for 24 h. Phosphorylation of JNK **(A)**, ERK **(B)** and p38 **(C)** was examined by Western blotting. Data represent the mean ± S.E.M. of four independent experiments. ^*^, *p* < 0.05 compared with the control group; ^#^, *p* < 0.05 compared with the VEGF-treated group.

The mitogen-activated protein kinase (MAPK) pathways regulate various biological functions of endothelial cells for angiogenesis [34, 35]. We evaluated whether the JNK, ERK and p38 protein kinases are involved in the anti-angiogenic effect of tanshinone IIA. As shown in Figure 4, stimulation of EPCs with VEGF significantly increased JNK phosphorylation, but not ERK and p38 phosphorylation. Tanshinone IIA dramatically suppressed VEGF-induced JNK activation in a concentration-dependent manner. These results demonstrate that the JNK signaling pathway is involved in tanshinone IIA-induced anti-angiogenic activity in human EPCs.

### Tanshinone IIA inhibits angiogenesis and the expression of progenitor cell marker *in vivo*

**Figure 5:**
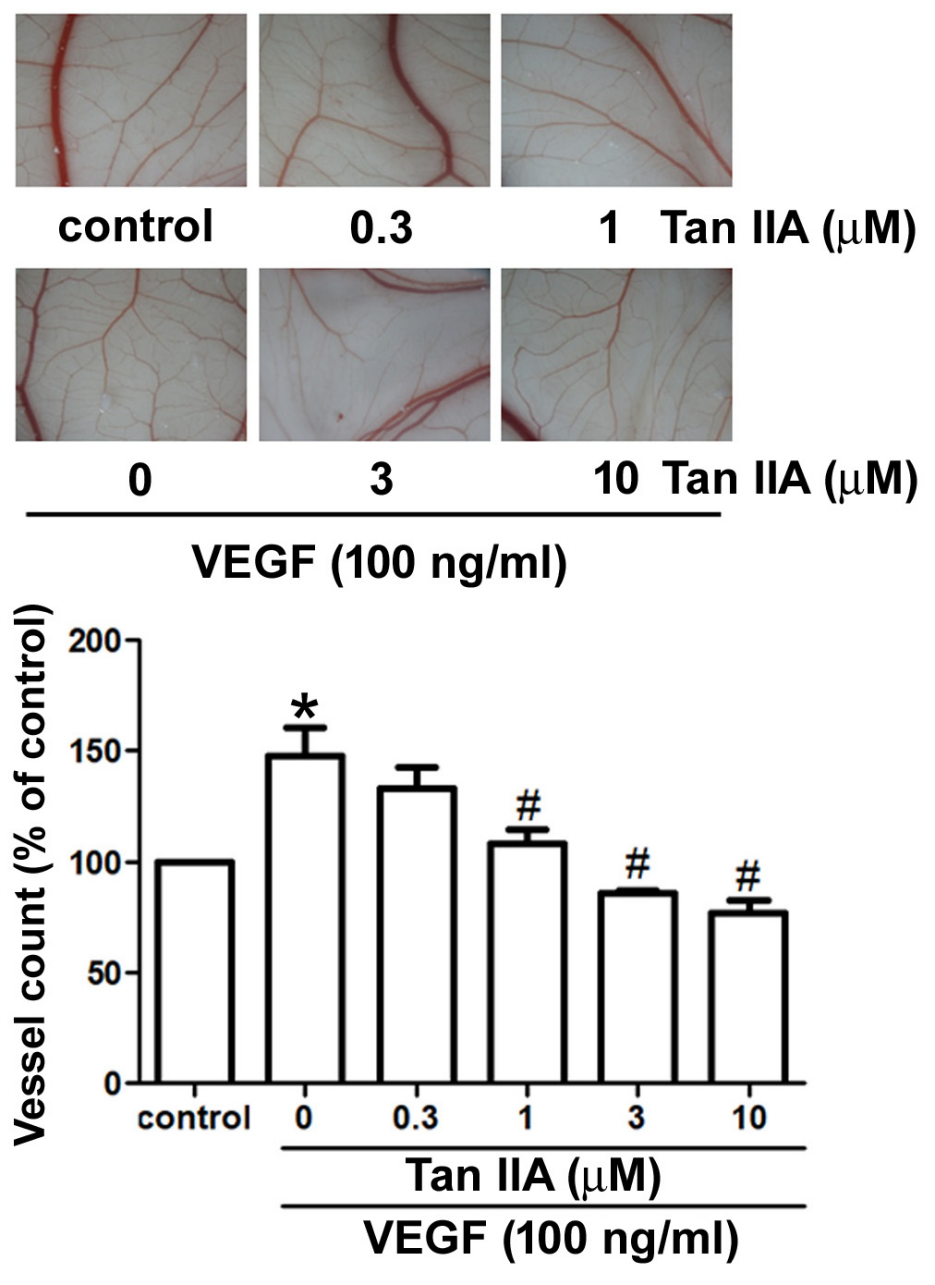
Effects of tanshinone IIA on VEGF-induced angiogenesis in the CAM model Five-day-old fertilized chick embryos were treated with or without VEGF (100 ng/ml) in the absence or presence of various concentrations of tanshinone IIA for 3 days. After treatment, the CAMs were examined by microscopy and photographed. Data represent the mean ± S.E.M. (N number ≥ 8 chick embryos per group). ^*^, *p* < 0.05 compared with the control group; ^#^, *p* < 0.05 compared with the VEGF-treated group.

**Figure 6:**
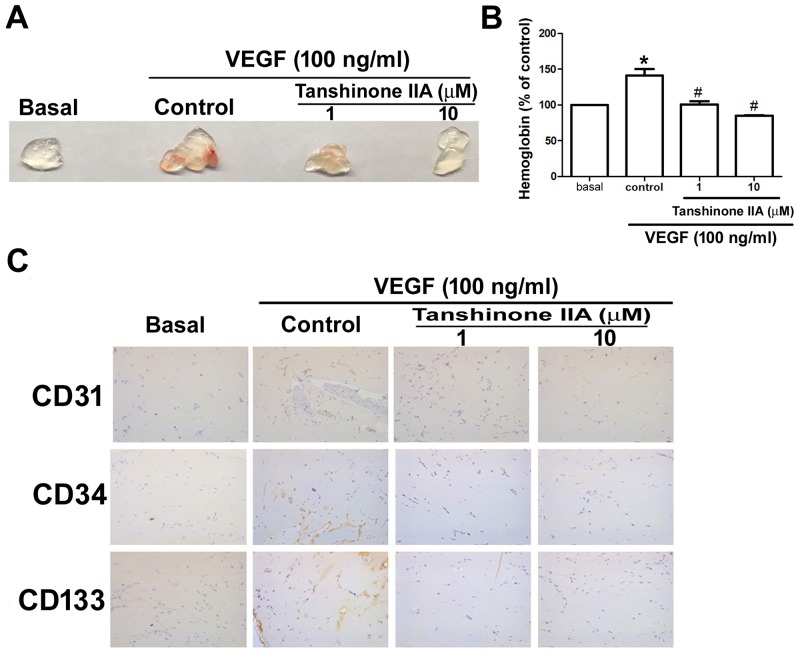
Effects of tanshinone IIA on *in vivo* angiogenesis and EPC marker expression in the Matrigel plug model Matrigel plugs were treated with PBS (basal group) or VEGF (control group) in the absence or presence of tanshinone IIA (1 and 10 μM) and subcutaneously injected into the flanks of nude mice (N number ≥ 8 mice per group). After 7 days, the plugs were photographed **(A)** and hemoglobin levels were quantified **(B)**. Specimens from the plugs were immunostained with antibodies against CD31, CD34 and CD133 **(C)**. Data represent the mean ± S.E.M. of at least 5 mice per group. ^*^, *p* < 0.05 compared with the basal group; ^#^, *p* < 0.05 compared with the control group.

We performed the *in vivo* CAM assay to characterize the effect of tanshinone IIA on angiogenesis. Whereas VEGF increased vessel formation in the CAM model, tanshinone IIA significantly repressed VEGF-enhanced vessel formation in a dose-dependent manner (Figure 5). The Matrigel implant assay in mice was used to confirm the *in vivo* anti-angiogenic activity of tanshinone IIA. We found that VEGF promoted microvessel formation and hemoglobin content in Matrigel plugs, whereas tanshinone IIA profoundly inhibited this process (Figure 6A and 6B). Immunohistochemical staining demonstrated the presence of the vessel marker CD31 and EPC-specific markers CD34 and CD133, all of which were visibly reduced by tanshinone IIA (Figure 6C). Taken together, these data reveal that tanshinone IIA impedes EPC-regulated angiogenesis *in vivo*.

## DISCUSSION

The metastatic cascade is a multi-step process encompassing local invasion of tumor cells into the adjacent tissue [23], transendothelial migration of cancer cells into vessels [36], angiogenesis [37] and proliferation [38]. Angiogenesis is the most critical step, as the growing tumor requires a supply of oxygen and nutrients to ensure its growth. The metastatic spread of tumors also depends on growth of the vascular network. Angiogenesis inhibitors represent a breakthrough in the treatment of cancer and age-related macular degeneration (AMD). [39]. Compelling evidence indicates that tumor angiogenesis is supported by the mobilization and functional incorporation of other cells such as EPCs [40]. A recent study demonstrates that EPCs promote early tumor growth and also late tumor metastasis by activating the angiogenic switch. In addition, EPCs contribute to neovessel formation in tumors by secreting abundant angiogenic growth factors during tumor development [41]. It has been reported that certain chemotherapy drugs can trigger the mobilization of circulating EPCs and their subsequent ‘homing’ into the tumor [42]. EPC-targeting therapies may therefore be a promising strategy to block angiogenesis-facilitated tumor growth and metastasis. In this study, we used EPCs to investigate the anti-angiogenic effects of tanshinone IIA. Our results showed that tanshinone IIA inhibited VEGF-induced migration and tube formation of human EPCs in a concentration-dependent manner. We also provide evidence showing that the anti-angiogenic effects of tanshinone IIA in EPCs are not related to cytotoxicity. Importantly, tanshinone IIA markedly suppressed *in vivo* angiogenesis and the expression of specific EPC markers in an animal tumor model. Based upon these findings, we suggest that tanshinone IIA may be a potential angiogenesis inhibitor that targets EPCs.

*Salvia miltiorrhiza* (Danshen) is a TCM product with a long history of good efficacy and tolerability in the treatment of cardiovascular diseases. Danshen and its preparations have been reported to be effective treatments for congestive heart failure and angina pectoris [16, 43]. Tanshinone IIA is the major active compound in Danshen, with documented cardiovascular bioactivity and safety [44]. Previous studies have shown that tanshinone IIA exhibits anti-angiogenic activity in HUVECs [21, 22]. In this study, we report for the first time that tanshinone IIA anti-angiogenic effects in human EPCs. Our data reveal that nontoxic concentrations of tanshinone IIA inhibit EPC angiogenesis *in vitro* and *in vivo*. Tanshinone IIA is a promising natural product worthy of further investigation for the treatment of angiogenesis-related diseases. Tanshinone IIA is the main constituent in Bushen Huoxue Qubi (BHQ) granules, a TCM preparation that is used clinically for the treatment of blood stasis syndrome. A pharmacokinetics and tissue distribution study involving rats with acute blood stasis treated with orally administered BHQ has reported significantly higher values for the area under the concentration-time curve (AUC), maximum plasma concentration (C_max_), biological half-life (t_1/2_), lower total body clearance (CL) and apparent volume of distribution (Vd) of tanshinone IIA in plasma and higher AUC_0-t_ of tanshinone IIA in the analyzed tissues of the BHQ-treated rats, compared with normal rats [45]. In addition, high density lipoprotein (HDL)-loaded tanshinone IIA markedly improved pharmacokinetic behaviors of tanshinone IIA *in vivo* [46]. Whether HDL-tanshinone IIA has greater anti-angiogenic effects than tanshinone IIA in EPCs needs further examination. Evidence indicates that tanshinone IIA (20 - 100 μM) induces osteosarcoma apoptosis [47]. This current study suggests that tanshinone IIA at low concentrations (0.3 - 10 μM) reduces EPC-associated angiogenesis, indicating that the effective dose of tanshinone IIA in EPCs has physiologically relevant antitumor activity.

The MAPK pathway plays a key role in VEGF-regulated angiogenesis [34]. Here, we found that VEGF only significantly promoted the phosphorylation of JNK, and not ERK and p38, in EPCs. Notably, tanshinone IIA concentration-dependently diminished VEGF-induced JNK phosphorylation, indicating that tanshinone IIA may suppress VEGF-dependent EPC angiogenesis through the JNK signaling pathway. Involvement of the PLC pathway has been documented in VEGF-dependent angiogenesis [31]. Akt signaling is also a critical mediator in EPC angiogenesis [32]. In this study, VEGF-induced PLC and Akt phosphorylation was dramatically reversed by tanshinone IIA in EPCs. These results suggest that the PLC and Akt pathways are probably both involved in tanshinone IIA-induced suppression of EPC angiogenesis. Whether any crosstalk exists between the PLC, Akt and JNK pathways after tanshinone IIA treatment deserves further investigation.

**Figure 7:**
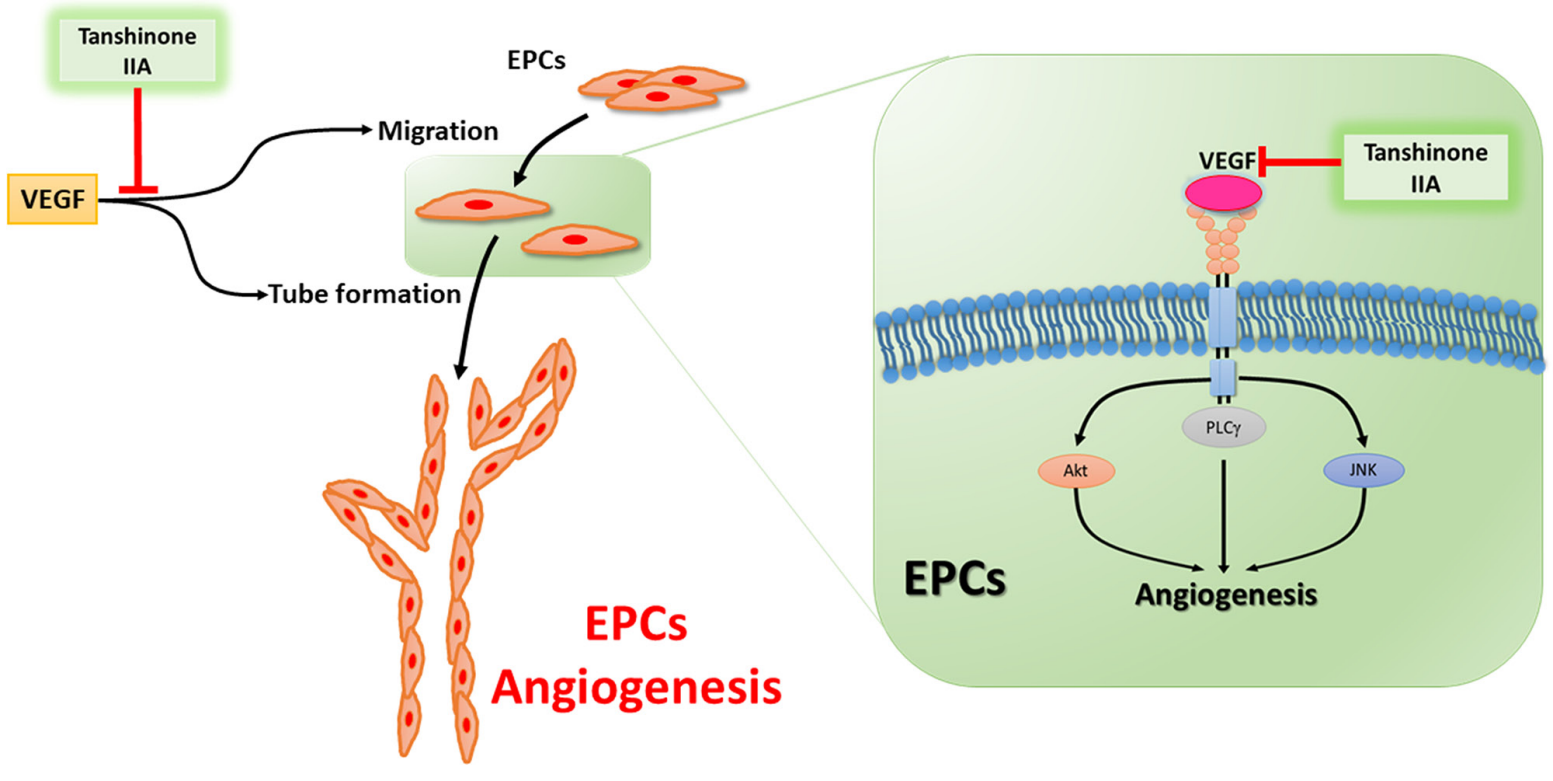
Schema of tanshinone IIA-induced anti-angiogenesis property in human endothelial progenitor cells Tanshinone IIA reduces EPCs angiogenesis *in vitro* and *in vivo* by controlling the PLC, Akt and JNK signaling pathways.

In conclusion, the current report discloses a novel mechanism by which tanshinone IIA reduces EPC angiogenesis *in vitro* and *in vivo*. We demonstrate that tanshinone IIA antagonizes EPC migration and tube formation by controlling the PLC, Akt and JNK signaling pathways (Figure 7). EPCs have been characterized as having the ability to dictate angiogenesis and metastasis in the tumor microenvironment. To our knowledge, this study is the first to identify that tanshinone IIA has anti-angiogenic activity against human EPCs. Our findings suggest that tanshinone IIA may serve as a lead candidate for further development of novel angiogenic inhibitors that are capable of blocking cancer progression and metastasis.

## MATERIALS AND METHODS

### Materials

Anti-CD133 antibody (Cat. No. orb13002) was obtained from Biorbyt (Biorbyt, Cambridge, UK). CD31 (Cat. No. ab9498) and CD34 (Cat. No. ab81289) antibodies were obtained from Abcam (Cambridge, MA, USA). Recombinant human VEGF was bought from PeproTech (Rocky Hill, NJ, USA). Anti-p-PLC (SC-136186), anti-p-Akt (SC-16646-R), anti-Akt (SC-5298), anti-p-ERK (SC-7383), anti-ERK (SC-154), anti-p-p38 (SC-166182), anti-p38 (SC-535), anti-p-JNK (SC-6254), anti-JNK (SC-7345), anti-mouse and anti-rabbit conjugated horseradish peroxidase antibodies were purchased from Santa Cruz Biotechnology (Santa Cruz, CA, USA).

### Cell culture

The study protocol for isolation of human EPCs was approved by the Institutional Review Board of Mackay Memorial Hospital, Taipei, Taiwan (reference number: 13MMHIS062). Peripheral blood mononuclear cells (PBMCs) were centrifuged from healthy donors using Ficoll-Paque PLUS solution (Amersham Biosciences, Uppala, Sweden), according to the manufacturer’s protocol. CD34^+^ progenitor cells were isolated from PBMCs using the CD34 MicroBead kit and MACS Cell Separation System (Miltenyi Biotec, Bergisch Gladbach, Germany), then cultured in MV2 complete medium containing MV2 basal medium and growth supplement (PromoCell, Heidelberg, Germany) supplemented with 20% defined fetal bovine serum (FBS) (HyClone, Logan, UT, USA) and maintained at 37°C in a humidified atmosphere of 5% CO_2_ [23, 24].

### Cell viability assay

EPCs were treated with or without tanshinone IIA for 24 h, then subjected to 3-(4,5-dimethylthiazol-2-yl)-2,5-diphenyltetrazolium bromide (MTT; 0.5 mg/mL) for 30 min. MTT was dissolved in dimethylsulfoxide (DMSO) and the absorbance was measured at 550 nm using a microplate reader (Bio-Tek, Winooski, VT, USA) [25].

### Cytotoxicity assay

EPCs were seeded onto 96-well plates in a density of 5 x 10^3^ cells per well. After 24 h incubation, cells were treated with MV2 complete medium containing 2% FBS in the absence or presence of tanshinone IIA. The percentage of LDH release in the collected medium was determined using a non-radioactive cytotoxicity assay kit (Promega, Madison, WI, USA).

### Western blot analysis

EPCs were lysed and processed by sodium dodecyl sulfate-polyacrylamide gel electrophoresis and transferred to Immobilon polyvinyldifluoride membranes. The blots were blocked with 4% bovine serum albumin then probed with primary antibodies, before being treated with donkey anti-rabbit peroxidase-conjugated secondary antibody. Finally, the blots were visualized by enhanced chemiluminescence using an ImageQuant LAS 4000 system (GE Healthcare, Pewaukee, WI, USA) [26].

### Measurement of EPC migratory ability

EPCs (5 x 10^4^ cells/well) were seeded onto the upper chamber with MV2 complete medium, then incubated in the bottom chamber with MV2 complete medium containing VEGF with the indicated concentrations of tanshinone IIA. After 24 h of treatment, cells on the upper side of the filters were mechanically removed, and those that had migrated to the lower side were fixed with 4% formaldehyde, then stained with 0.05% crystal violet. Cells on the upper side of the Transwell membrane were photographed and counted under a microscope [27].

### Measurement of EPC tube formation

EPCs (3 × 10^4^ cells) were seeded onto pre-coated Matrigel plates (BD Biosciences, Bedford, MA, USA) in MV2 complete medium containing VEGF with the indicated concentrations of tanshinone IIA, followed by incubation for 24 h at 37°C. EPC tube formation was photographed and numbers of tube branches were calculated using MacBiophotonics Image J software [27, 28].

### Chick chorioallantoic membrane (CAM) assay

VEGF and the indicated concentrations of tanshinone IIA were mixed with Matrigel and applied into the center of the developing chorioallantoic membrane of a fertilized chicken egg. After 3 days, CAMs were collected for microscopy and photographic documentation. Angiogenesis was quantified by counting the number of blood vessel branches; at least 10 viable embryos were tested for each treatment.

### Matrigel plug assay

Four-week-old nude male mice were subcutaneously injected with 300 μl Matrigel containing VEGF with the indicated concentrations of tanshinone IIA. The plugs were collected after 7 days and then fixed, embedded in paraffin, and processed by immunohistochemical staining for CD31, CD34 and CD133 antibodies [29]. Hemoglobin content was examined using Drabkin’s reagent. All animal procedures were performed according to approved protocols issued by the China Medical University (Taichung, Taiwan) Institutional Animal Care and Use Committees.

### Statistical analysis

Data are presented as the mean ± standard error of the mean. Statistical analysis of comparisons between 2 samples was performed using the Student’s *t* test. Statistical comparisons of more than 2 groups were performed using one-way analysis of variance with Bonferroni’s post-hoc test. In all cases, *p* < 0.05 was considered statistically significant.

## SUPPLEMENTARY MATERIALS FIGURES


