# Supplementary material for: Tanshinone IIA inhibits angiogenesis in human endothelial progenitor cells in vitro and in vivo
Source: Oncotarget. 2017 Nov 24;8(65):109217–27. doi: 10.18632/oncotarget.22649 (PMC5752515; doi:10.18632/oncotarget.22649)
Supplement: Supplementary file 1 [file oncotarget-08-109217-s001.pdf]

Tanshinone IIA inhibits angiogenesis in human endothelial progenitor cells *in vitro* and *in vivo*

SUPPLEMENTARY MATERIALS

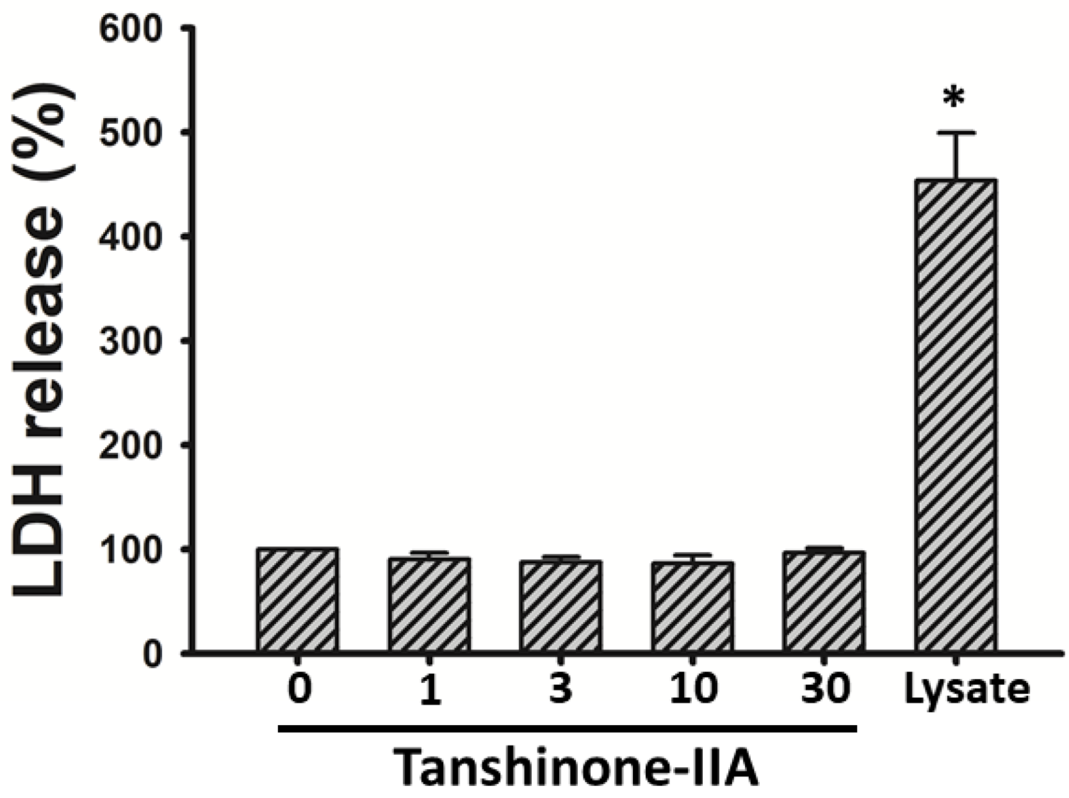

Supplementary Figure 1: Effect of tanshinone IIA on cytotoxicity in human EPCs under long-term treatment. EPCs were incubated with the indicated concentrations of tanshinone IIA for 48 h, then cytotoxicity was determined using LDH assay. Data represent the mean ± S.E.M. of four independent experiments. *, $p < 0.05$ compared with the control group.

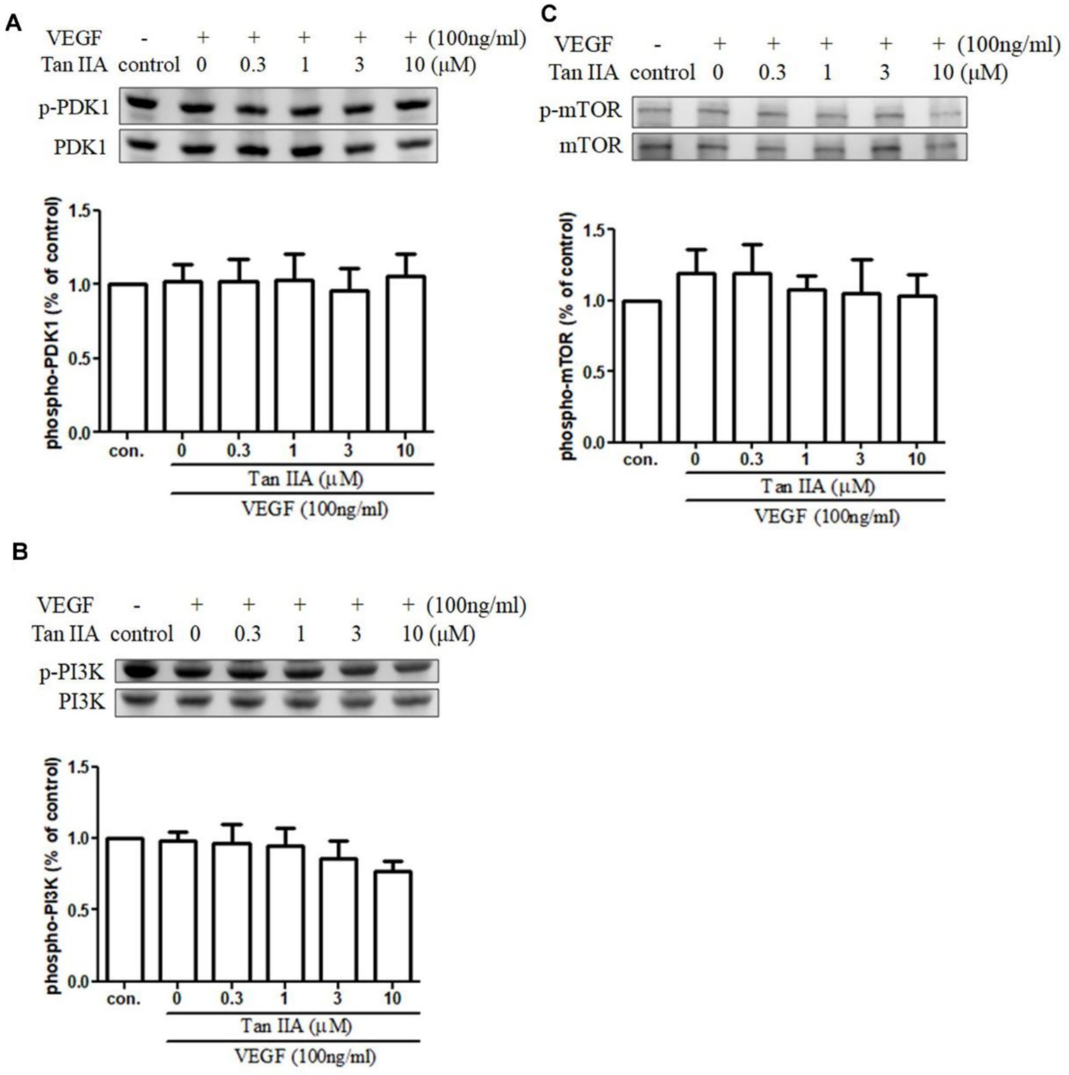

Supplementary Figure 2: Effect of tanshinone IIA on the PDK1, PI3K and mTOR pathways in human EPCs. EPCs were incubated with VEGF-A (100 ng/ml) and the indicated concentrations of tanshinone IIA for 24 h. Then, the phosphorylation of PDK1 **(A)**, PI3K **(B)** and mTOR **(C)** was examined by Western blotting. Data represent the mean ± S.E.M. of three independent experiments.
